# Cytogenetic culture failure and its causes in hematological disorders; a single centre perspective

**DOI:** 10.1186/s13039-022-00635-9

**Published:** 2023-02-10

**Authors:** Sarah Javed, Jawad Hassan, Maliha Naz, Saira Shan, Madiha Abid, Tahir Sultan Shamsi

**Affiliations:** 1 Department of Clinical and Molecular Cytogenetics, National Institute of Blood Disease and Bone Marrow Transplantation (NIBD), ST 2/A, Block 17, Gulshan-E-Iqbal, KDA Scheme 24, Karachi, 75300 Pakistan; 2 Research Department, NIBD, Karachi, Pakistan; 3 Department of Clinical Hematology, NIBD, Karachi, Pakistan

**Keywords:** Cytogenetic analysis, Culture failure, Hematological disorders, Metaphases, Bone marrow aspirate

## Abstract

**Objective:**

To highlight the reasons of culture failure in bone marrow aspirate samples sent for Cytogenetic analysis and to identify the associated parameters causing this impact.

**Methodology:**

This is a retrospective cross-sectional study conducted in the Clinical and Molecular Cytogenetics Laboratory of NIBD Hospital, Karachi, Pakistan. The rates of culture failure are assessed from the year 2017–2020 along with their reasons. Bone Marrow aspirate samples of patients with hematological malignancies were cultured for chromosomal analysis, both at the time of diagnosis or relapse. Statistical analysis was performed using SPSS version 25.

**Results:**

A total of 1061 bone marrow aspirate samples were assessed for cytogenetic culture failures from the duration of 2017 to 2020. Ratio of males was predominantly higher i.e. 62.7% than female 37.3% with Mean ± SD age was 36.78 ± 18.94. Frequency of culture failure in the year 2020 was relatively high 20% as compared to the preceding years i.e. 8% in 2017, 6% in 2018, 7% in 2019. However, the patients were diagnosed with the following hematological malignancies; ALL 23%, CML 17.1%, AML 16.5% and AA 12.5%. Among the reasons of culture failure, cytogenetic analysis of patients with on-going chemo resulted in significant culture failures with p-value < 0.001 and the hematological malignancy, Acute Promyelocytic Leukemia, significantly impacted the growth of bone marrow aspirate cultures, with p-value < 0.001.

**Conclusion:**

Significant findings were associated with causative factors of culture failure including on-going treatment and sample issues of clotted bone marrow as well as with the clinical diagnosis. These evaluations facilitated in overcoming the rise in culture failures. As per our knowledge, no such data, discussing the effects of various parameters such as sample quality, diagnosis, effects of treatment etc., has been documented previously.

## Introduction

Cytogenetic studies constitute a major part in predicting the outcome of a treatment for certain hematological disorders. Hematological disorders are the neoplasms that develop in bone marrow-derived cells. Many patients with leukemia, lymphoma, or other malignant hematologic diseases have malignant cells that have acquired clonal chromosomal aberrations [6]. Nowadays, cytogenetic testing is considered to be a requirement for diagnosing hematologic malignancies. Recurrent structural abnormalities are useful diagnostic and prognostic indicators for various diseases, and they also help with the choice of prescription treatments for personalized oncology [1].

The chromosomal arrangement of the bone marrow sample can be analyzed using the conventional cytogenetic analysis techniques. For cytogenetic research worldwide, chromosome banding has long been the gold standard because of its high success rate in identifying crucial copy number variation and structural variation known to trigger and drive disease progression [25]. These techniques are better than molecular analyses and FISH because they are able to provide unique and atypical chromosomal rearrangements. However, it is more challenging and time-consuming due to the difficulty in extracting cells from bone marrow, depending on the disease-state of the patient [1]. In-vitro culturing of bone marrow sample is the method of choice for providing the clinicians with a treatment regimen. It plays a vital role in assessing the prognostic level of the hematological malignancy by analyzing good quality metaphase spreads to determine the presence of any clone according to the particular diagnosis. The unavailability of analyzable and reportable metaphases in a patient’s sample referred for cytogenetic analysis, is considered as culture failure [19].

Not all cultures, even after providing the same nutrient mediums and growth factors simultaneously, yield sufficient number of analyzable metaphase chromosomes and hence fail to exhibit any result [19]. One of the important factors that commonly deter the success of a cytogenetic culture is the quality of bone marrow sample i.e. the clotting of bone marrow sample, irrespective of the disease. For instance, samples of different patients with similar diagnosis can give completely different chromosome morphology, even when processed simultaneously. In short, some samples are able to grow well in culture and yield good quality chromosomes while others defy all the tricks, a Cytogeneticist can apply, and still give poor, unanalyzable and small metaphase chromosomes [7, 19].

Some of the other factors that impact the culture growth, include; hypocellularity, low proliferative rate in tissue culture, insufficient number of metaphases, less viability of cells, disease and the treatment induced cytotoxicity [7]. The failure rate for bone marrow and neoplastic blood specimen cultures should not exceed 10% [4]

Due to an upsurge of culture failures in 2020, this study was planned in order to document our laboratory experiences and the troubleshooting that was done to prevent these recurring issues. Culture failure in cytogenetics has not been studied previously and literature on this topic is scarce therefore, this study is done to highlight the main issues pertaining to culture failures.

## Materials and methods

A retrospective observational study on reasons and rates of culture failure was assessed in the Clinical and Molecular Cytogenetics Department in the year of 2017 to 2020 after the approval of Institutional Review Board (IRB# 232/20–2021), of NIBD Hospital.

A total of 1061 samples were processed from pre-analytical to analytical phase as per the standard protocol for cytogenetic analysis [8, 12] involving un-stimulated 24 h and PHA/Interleukin-stimulated 72–96 h cultures as per the patient’s diagnosis. After the metaphase arrest, the samples were proceeded for harvesting involving hypotonic treatment and fixation of cells. Slides were prepared and Giemsa-banded for observing metaphase spreads, if present [8]. Patients with the following hematological disorders were included in the study,Aplastic Anemia (AA, Acute Lymphoblastic Leukemia (ALL, Myeloproliferative Neoplasm (MPN, Acute Myeloid Leukemia (AML, Myelodysplastic Syndrome (MDS, Acute Promyelocytic Leukemia (APML, Lymphoproliferative disorder (LPD, diagnosed on the basis of Bone Marrow Biopsy.

Statistical analysis was performed on SPSS version 25. Frequency and percentages were computed for categorical variables such as age and gender and mean and standard deviation were estimated for quantitative variables including culture failure with respect to different parameters including sample quality, sample issues such as high cell count or low cell count, effect of treatment, diagnosis etc.

## Results

**Fig. 1:**
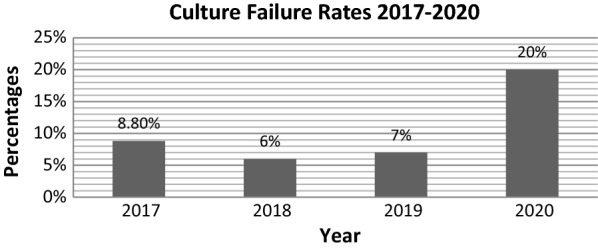
Culture failure percentile over the past years where the year 2020 showed a sudden peak (20%) in culture failures

A total of 1061 participants were recruited in this study for the assessment of cytogenetic culture failures. Out of these, 665 (62.7%) were male and 396 (37.3%) were female with Mean ± SD age (year) of 36.76 ± 18.92. As per the statistical analysis of our data, in the year 2020 highest number of culture failures were recorded i.e. 76(20%) followed by 2017 with 20(8.8%), 2019 with 17(7%) and the lowest were recorded in the year 2018 with 12(6%) as presented in Fig. 1. No significant findings of culture failure were associated with age and gender.

The most commonly reported hematological malignancies included ALL 241(22.7%), CML 182(17.1%), AML 176(16.5%) and AA 133(12.5%) from 2017 to 2020. Out of the total, 125 (11.7%) culture failures were observed from 2017 to 2020.

**Fig. 2:**
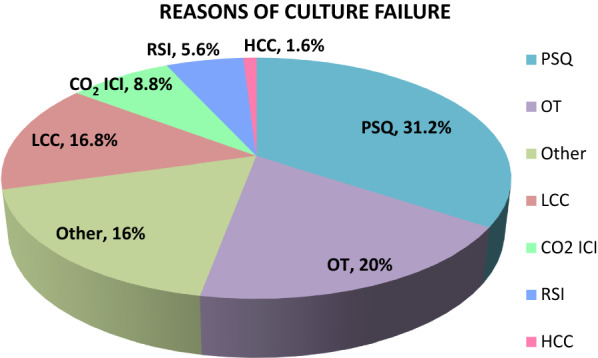
Overall reasons of Culture Failure from 2017–2022 (where *RSI* reagent supply issue, *HCC* high cell count, *PSQ* poor sample quality, *OT* on treatment, *LCC* low cell count, *CO*_2_ *ICI* CO_2_ incubator issues)

Reasons associated to certain parameters such as bone marrow clotting in sample, on-going treatment, low & high cell counts along with other issues; were analyzed with respect to culture failure as displayed in Fig. 2.

A significant association was reported among causative factors with respect to the culture failure in the year 2020, as shown in Table 1 with p-value i.e. < 0.001.

**Table 1:** Association of culture failure in 2020 with respect to different reasons

Reasons	No. of culture failures (%)	*P* value
OT	30.2	0.001
PSQ	19.7	
LCC	17.1	
CO_2_ ICI	14.4	
RSI	9.2	
Other	9.2	

*PSQ* poor sample quality, *OT* on treatment, *LCC* low cell count, *CO*_2_ *ICI* CO_2_ incubator issues, *RSI* reagent supply issues

Frequency of culture failure was noted from 2017 to 2020 with respect to particular hematological malignancy as shown in Table 2.

**Table 2:** Association of culture failure with diagnoses

Diagnosis	Culture failure (%)	P-value
LPD	17.4	0.001
ALL	10.7	
AML	11.9	
APML	36.3	
MPN	5.7	
MDS	16.3	
ITP	10.3	
AA	9.7	
PV	20	
Anemia	13.9	

*ALL* acute lymphoblastic leukemia, *LPD* lymphoproliferative disorder, *AML* acute myeloid leukemia, *APML* acute promyelocytic leukemia, *MPN* myeloproliferative neoplasm, *MDS* myelodysplastic syndrome, *ITP* immune thrombocytopenia, *AA* aplastic anemia, *PV* polycythemia vera

In Table 2, we have categorized the hematological malignancies as MPN (which includes CML: Chronic Myeloid Leukemia, ET: Essential Thrombocythemia and MF: Myelofibrosis) LPD (which consists of PCD: Plasma Cell Disorder, MM: Multiple Myeloma, CLL: Chronic Lymphocytic Leukemia and Lymphoma).

**Fig. 3:**
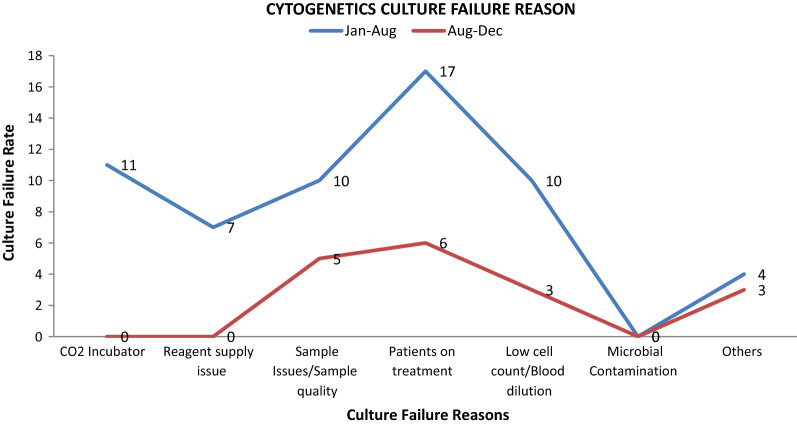
Comparative graphical representation of culture failure rates from **a** January-August 2020 (Blue Line) and **b** from August–December 2020 (red line)

However, the high peak observed in the culture failure rate in the year 2020 as shown in Fig. 3 was further investigated to elaborate the factors that affected the growth of cultures.

## Discussion

This study was done to evaluate the possible reasons for the upsurge of culture failure rate in the year 2020. The number of culture failure cases increased up to 10% from the preceding years. After analyzing the causative factors of culture failure through statistical analysis, particularly in the year 2020, significant contributing factors primarily affecting the cytogenetic culture growth were found to be poor bone marrow sample quality (i.e. samples with high cell count forming a visible clot) and the patients who came immediately after their chemo induction cycles. Secondly, the association of culture failure with respect to the diagnosis revealed Acute Promyelocytic Leukemia to be poor growing hematological disorder. No substantial association has yet been noted to date, with regards to cytogenetic culture failures associated with these particular parameters and diagnosis.

As per Mitelman et al. bone marrow suppression prominently occurs as a result of chemotherapy induction cycles which in turn causes little to no growth in cultures for cytogenetic analysis (2015). In this study, out of 76 culture failure in the year 2020, 30% samples failed to grow in-vitro due to the patient’s on-going or recently completed treatment. Keeping this in regard, the clinicians were requested not to send bone marrow samples immediately after treatment for cytogenetic analysis. This led to a significant reduction in culture failure rates in second half of year 2020, visible by statistical analysis (Fig. 3, red line). Recently undertaken chemotherapy may decrease the rate of culture success by decreasing the number of cells at metaphase stage, as immature cells are most often eliminated [5]. Some of the contributing factors that affect the growth of cultures in cytogenetics include bone marrow sample quality, improper sampling technique for bone marrow, delay in sample transit, internal laboratory errors, specimen type, suppression due to disease-condition and effects of treatment on culture growth [7, 13, 20]. An important issue to note is the proper heparinization of the sample and maintaining the anticoagulant to sample ratio because if the ratio gets disturbed or inappropriate anticoagulant is used the sample becomes nonviable with time and a clot starts to form which entraps all the cells needed for a cytogenetic study, resulting in no growth and hence culture failure [7]. Often, due to lack of proper expertise, a later portion of the marrow sample is sent to the laboratory which is heavily contaminated with blood and does not have effectively dividing cells [14]. It has been observed that bone marrow samples of myeloid disorders may tolerate delay to an extent unlike the lymphoid disorder samples [11]. In order to avoid clotting of bone marrow samples sent for cytogenetic analysis, it is recommended to properly train the MDS perfoming the Bone Marrow aspiration procedure.

In addition to all the aforementioned issues regarding the culture failure of a bone marrow sample another important issue that can affect in-vitro cell growth is the leukemic state of the patient. It inflicts changes in the bone marrow which in turn affects hematopoiesis as well as the way in which bone marrow cells are produced [2, 16]. For instance, even with normal blood counts, hematopoietic impairment persists long in the patients who are long-term survivors of AML and it does not improve with the passage of time therefore, making it problematic for cytogenetic analysis and hence reporting [15]. Significant in-vitro cell death can be a problem in Acute Lymphoblastic Leukemia (ALL) or Lymphoma therefore, multiple cultures are recommended. Acute Lymphoblastic Leukemia might affect the lymphoid cells due to which they are incapable of responding to mitogenic reagents or the treatment may have suppressed the immune response resulting in culture failure [10].

Bone marrow is particularly prone to chemotherapy damage as it exhibits severe cytotoxic effects. The patients who receive multiple rounds of chemotherapy repeatedly show irreversible bone marrow damage which compromises the hematopoietic reserves and then in turn affects its function. Almost all chemo medicines suppress the bone marrow and the duration of this effect differs according to the dosage and the medicines used for the treatment. Higher the dose, much deleterious effects will be observed in the cytogenetic cultures [8] (Berger 1983; Keinanen 1986). All these issues related to bone marrow usually recuperate within 6 weeks post-treatment, but it can take longer after strong chemotherapy regimens or combination of drugs [21, 23, 24]. In order to get the follow-up information regarding a patient’s known marker such as a previously reported translocation or any other abnormality, Fluorescent in-situ Hybridization (FISH) studies can be performed on the bone marrow smear. However, FISH studies can only be used for a particular known anomaly and would not provide any further information concerning the additional chromosomal aberrations.

The parameters like CO_2_ incubator issues and the untimely reagent supply might be the concerns of developing countries more than the Western countries. Minimizing these internal laboratory issues by providing optimal CO_2_ provision in an alternate CO_2_ incubator for in-vitro cell culture of bone marrow samples and the timely reagent supply can reduce the rates of culture failure effectively. Failure of cytogenetics culture to grow in-vitro is most often an inevitable challenge a cytogeneticist has to face from time and again. Although some cultures are just unable to grow no matter the amount of nutrients provided and all quality measures taken but sometimes, even minor issues can cause trouble and hence the on-set of culture failure begins. As much as we worry about the failures of cultures and take every preventive measure in handling the samples and culturing them at optimal conditions while providing the effective nutrients and growth serums required for the cell growth, some issues arise even before the sample is treated in the cytogenetics laboratory [9, 17, 18].

Our current study and its findings were limited to a single centre hence the small sample size and the literature on cytogenetics culture failure and its reasons is scarce as well.

## Conclusion

Cytogenetic studies have always been a golden choice for clinicians around the globe in order to evaluate the prognosis for a certain hematological malignancy and emphasizing to minimize the relapse rate accordingly. The diagnosis and clinical treatment of patients, as well as the discovery of the genomic basis of the pathophysiology of these diseases, have all benefited greatly from the cytogenetic investigation of hematological malignancies. The most notable achievements of cytogenetics in human cancer are unquestionably leukemias and lymphomas, which have adapted themselves well to karyotypic investigation. A number of chromosomal alterations have been demonstrated to have substantial prognostic value as well and are currently being explored as quantifiable targets for therapeutic response, with the help of conventional cytogenetics. However, the necessity for in-vitro cell division is a drawback of chromosomal banding. In order to perform a chromosomal banding study in some hematological malignancies, a considerable number of analyzable metaphase cells may not be acquired in culture, resulting in culture failure. In this study, some of the major issues related to the emergence of culture failures in cytogenetics have been highlighted and investigated. Even after analyzing these parameters a cytogeneticist can never predict when a culture will fail to exhibit results and defy all the probabilities of achieving a good outcome.

## Data Availability

All the dataset that supports the conclusion of this research study has been incorporated within the article.
